# Promising trends and influencing factors of complementary feeding practices in Côte d'Ivoire: An analysis of nationally representative survey data between 1994 and 2016

**DOI:** 10.1111/mcn.13418

**Published:** 2022-09-07

**Authors:** Nan Dou, Evaniya Shakya, Raphia M. Ngoutane, Denis Garnier, Oka R. Kouame, Anne‐Sophie L. Dain, Aashima Garg, Stephen R. Kodish, Laura E. Caulfield, Laura E. Murray‐Kolb, Muzi Na

**Affiliations:** ^1^ Department of Nutritional Sciences, College of Health and Human Development The Pennsylvania State University University Park Pennsylvania USA; ^2^ Nutrition Department UNICEF Abidjan Côte d'Ivoire; ^3^ National Nutrition Programme, Ministry of Health and Public Hygiene Abidjan Côte d'Ivoire; ^4^ West and Central Africa Regional Office UNICEF Dakar Senegal; ^5^ UNICEF Headquarters New York New York USA; ^6^ Department of International Health, Bloomberg School of Public Health Johns Hopkins University Baltimore Maryland USA; ^7^ Department of Nutrition Science, College of Health and Human Sciences Purdue University West Lafayette Indiana USA

**Keywords:** complementary feeding, Côte d'Ivoire, Demographic and Health Surveys, Multiple Indicator Cluster Surveys, multivariable logistic regression models, risk factor analysis, trends

## Abstract

Poor complementary feeding (CF) challenges early childhood growth. We examined the trends and influencing factors of CF practices among children aged 6–23 months in Côte d'Ivoire. Using data from Demographic and Health Surveys (DHS, 1994–2011) and Multiple Indicator Cluster Surveys (MICS, 2000–2016), the trends and predictors of World Health Organization‐United Nations International Children's Emergency Fund CF indicators including the timely introduction of complementary foods (INTRO), minimum meal frequency (MMF), minimum dietary diversity (MDD) and minimum acceptable diet (MAD) were determined. Using 2016 MICS data, we applied multivariate logistic regression models to identify factors associated with CF indicators. Between 1994 and 2016, the mean proportion of children aged 6–8 months achieving INTRO was 56.9% and increased by about 25% points since 2006. Over 2011–2016, the proportion of children aged 6–23 months meeting MMF, MDD and MAD increased from 40.2% to 47.7%, 11.3% to 26.0% and 4.6% to 12.5%, respectively. Older children and those from urban households had higher odds of meeting MDD and MAD. Maternal TV watching was associated with higher odds of meeting MDD. The secondary or higher education levels of mothers significantly predicted higher odds of meeting INTRO and MDD. Currently, breastfeeding was also positively associated with odds of meeting MMF and MAD. Children from poorer households had lower odds of meeting MMF, MDD and MAD. Despite the improvements, CF practices remain suboptimal in Côte d'Ivoire. Influencing factors associated with CF were distributed across individual, household and community levels, calling for future programmes and policies to implement multi‐level strategies to improve young children's diet in Côte d'Ivoire.

## Key messages

- Over 2006–2016, the proportion of children aged 6–23 months meeting complementary feeding (CF) indicators, including introduction of complementary foods, minimum dietary diversity, minimum meal frequency and minimum acceptable diet (MAD), improved steadily.
- Suboptimal CF practices remain a concern, with 12.5% of children aged 6–23 months meeting MAD in 2016.
- Inappropriate CF practices are significantly associated with factors at individual (child age, maternal education TV watching and currently breastfeeding), household (wealth and urbanicity) and community levels (community‐level access to health care).

## Graphical abstract

Over 2006–2016, the proportion of children aged 6–23 months meeting complementary feeding (CF) indicators, including introduction of complementary foods, minimum dietary diversity, minimum meal frequency and minimum acceptable diet, improved steadily. Poor CF practices were significantly associated with influencing factors at the individual, household, and community levels, including older child age, maternal education levels, maternal TV watching, currently breastfeeding, residing in an urban area, wealthier households and living in communities with better health care access. Future programmes and policies are needed to implement multi‐level strategies to improve young children's diet in Côte d'Ivoire.

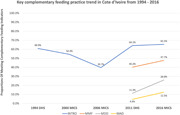

## 1 INTRODUCTION

Côte d'Ivoire has achieved successful progress in reducing child malnutrition but is still experiencing a high prevalence of stunting (United Nations Children's Fund World Health Organization & The World Bank, 2021). From 2000 to 2020, the proportion of stunted children under 5 years of age decreased from 33.6% to 17.8% (United Nations Children's Fund et al., 2021). Côte d'Ivoire experienced economic and political instability during civil wars and, since 2012, economic rebuilding and growth (The World Bank, 2021). War conflicts and economic instability exacerbated socioeconomic hardships (Fürst et al., 2010), posing great challenges for child nutrition.

Adequate child feeding practices play an important role in maintaining nutritional status among infants and young children (Black et al., 2013; Stewart et al., 2013). Exclusive breastfeeding is recommended by the World Health Organization (WHO) for all infants younger than 6 months of age (WHO, 2003). At 6 months, the introduction of other nutrients‐ and energy‐dense foods and liquids is critical for infants and young children (WHO, 2010). According to WHO, appropriate complementary feeding (CF) practices require the timely introduction of complementary foods with adequate frequency and diversity (WHO, 2010). Prior evidence revealed that appropriate CF practices benefit physical growth (Lassi et al., 2013) and neurocognitive development (Morley & Lucas, 1997; Prado et al., 2019) in childhood, and prevent chronic conditions in adulthood (D'Auria et al., 2020). In Côte d'Ivoire, however, CF practices are thought to be poor. In 2016, only 13.6% of children aged 6–23 months were fed with a minimum acceptable diet (MAD) according to recommended CF practices (UNICEF, 2020).

Nationally representative surveys, including the WHO Infant and Young Child Feeding (IYCF) module to assess CF, have been implemented in Côte d'Ivoire in the past 20 years; however, changes in CF practices over time and its related influencing factors have not been studied. Understanding the trends and risk factors of CF, described as the underlying determinants of child malnutrition by the UNICEF framework, is an appropriate first step to address child malnutrition (United Nations Children's Fund, 2020b). It is also essential to identify the modifiable risk factors related to CF practices to plan and implement effective interventions by targeting at‐risk individuals, households and communities (Stewart et al., 2013). Using nationally representative data between 1994 and 2016 from Côte d'Ivoire, this study aims to (1) describe the trends of CF practices in Côte d'Ivoire using the WHO‐defined CF indicators, and (2) explore influencing factors in the individual, household and community levels that predict CF practices in Côte d'Ivoire.

## 2 METHODS

### 2.1 Study sample

To understand the CF trends (aim 1), we extracted data on the key CF indicators from five nationally representative survey reports: the 1994 and 2011 Côte d'Ivoire Demographic and Health Surveys (DHS) reports and 2000, 2006 and 2016 Côte d'Ivoire Multi‐Indicator Cluster Survey (MICS) reports. Four independent researchers conducted data extraction in pairs. Any discrepancy (i.e., inaccurate data extracted from wrong tables in reports) between the researchers were resolved through group discussion until consensus was reached.

To explore the current influencing factors of CF (aim 2), we analyzed the most recent 2016 MICS data. Information on the 2016 MICS survey methodology, sampling procedure and questionnaires has been published previously (Institut National de la Statistique et al., 2016). Briefly, eligible women and children were included based on a two‐stage stratified sampling procedure. At the first stage, a total of 512 census enumeration areas were selected with probability as the primary sampling units (PSUs). At the second stage, 25 households were selected by systematic sampling within each PSU. Based on prior studies looking at CF practices in low‐ and middle‐income countries (Na, Aguayo, Arimond, Dahal, et al., 2018; Na, Aguayo, Arimond, Mustaphi, et al., 2018; Na et al., 2017), the inclusion criteria of mother–child pairs to be included in our analysis were: (1) mothers were between 15 and 49 years of age; (2) the youngest singleton child was aged 6–23 months; (3) children were alive at the time of the survey; and (4) children lived with their mothers. We included mothers aged 15–49 years old to decrease the possibility of enroling children with potential health problems born from teenage (<15 years) or older (>49 years) mothers. The study further defined the youngest singleton children aged 6–23 months to avoid potential recall bias and to prevent enroling more than one child from each household. In addition, only alive children living with their mothers were included, so the surveys were able to collect their CF practices from mother–child pairs.

### 2.2 CF practices

Four CF indicators defined by WHO in 2010 were analyzed in this study, including the introduction of solid, semisolid, or soft foods (INTRO), minimum meal frequency (MMF), minimum dietary diversity (MDD) and MAD (World Health Organization, 2010). Data for INTRO were available in 1994 and 2011 DHS and 2000, 2006 and 2016 MICS reports. Data for MMF, MDD and MAD were only available in the 2011 DHS and 2016 MICS reports. The CF indicators were defined per WHO definitions as follows (WHO, 2010):

*INTRO*: The proportion of infants 6–8 months of age who received solid, semisolid and soft foods in the previous day or night.

*MMF*: The proportion of breastfed and nonbreastfed children 6–23 months of age, who received solid, semisolid or soft foods (including milk for nonbreastfed children) the minimum recommended number of times or more in the previous day or night. For breastfed children, MMF is met if at least two solid/semisolid feeds occurred for children aged 6–8 months and at least three feeds occurred for children aged 9–23 months. For nonbreastfed children, MMF is met if at least four feeds of complementary food or milk for children aged 6–23 months occurred.

*MDD*: The proportion of children 6–23 months of age who received foods from four or more food groups in the previous day or night out of the following seven food groups: (1) grains, roots and tubers, (2) legumes and nuts, (3) dairy products, (4) flesh foods, (5) eggs, (6) vitamin‐A‐rich fruits and vegetables and (7) other fruits and vegetables.

*MAD*: The proportion of children 6–23 months of age who received the minimum recommended dietary diversity and the minimum recommended meal frequency in the previous day or night. For breastfed children, they are classified as having a MAD when they meet the MMF and MDD standards. For nonbreastfed children, they are classified as having a MAD when they meet the MMF standards and receive at least two milk feedings along with at least four food groups other than milk products.

### 2.3 Influencing factors

The selection of the influencing factors at the individual, household and community levels was based on the conceptual framework developed by Stewart et al. (2013) and our previous work in South Asia (Na, Aguayo, Arimond, Dahal, et al., 2018; Na, Aguayo, Arimond, Mustaphi, et al., 2018; Na, Aguayo, Arimond, Narayan, et al., 2018; Na et al., 2017). Individual‐level factors included child, maternal and paternal characteristics. For children, the following variables were included: sex, age, birth order, birth interval, measured birthweight, perceived birthweight and child morbidity including diarrhoea, fever and cough. Maternal characteristics included age, smoking status, education, marital status, occupation, nutritional status (height and body mass index), breastfeeding practices, utilization of reproductive health care, exposure to media and women's attitude towards domestic violence. Paternal characteristics included age and education. Household‐level factors included household structures and socioeconomic status. The household characteristics included the place of residence, sex of household head, number of household members, number of children under five years, types of cooking fuel, water characteristics (source and location of drinking water, time to get to water sources) and quintiles of overall household wealth index (higher quintiles indicate poorer households). The community‐level factor described access to health care within the community where the selected subjects lived. Based on the utilization of maternal and child nutrition and health care services among all respondents, the rank score for community access to health care was generated first and then categorized into quintiles. Higher rank scores or quintiles indicate poorer community access to health care. A detailed description of the factors is available elsewhere (Na et al., 2020).

### 2.4 Statistical analysis

All data analysis was performed using STATA/SE 15.1 (StataCorp). The prevalence of four CF indicators was extracted from the national DHS and MICS reports. Multivariable models were applied to determine the associations between influencing factors and CF indicators among children aged 6–23 months: (1) the bivariate associations between influencing factors and CF indicators were examined first to select the significant risk factors at *p*= 0.1, and (2) the selected variables from the bivariate analysis were included in the multivariate risk factor analysis.

## 3 RESULTS

**Figure 1:**
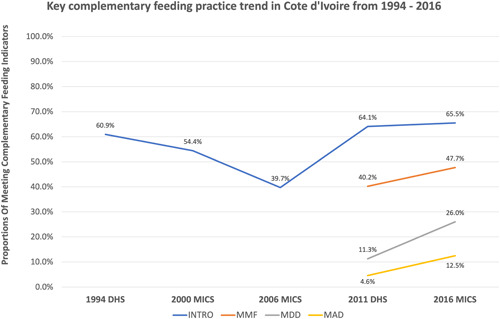
Trends of INTRO, MMF, MDD and MAD in Côte d'Ivoire from 1994 to 2016 (data presented in this figure were extracted from five nationally representative survey reports: the 1994 and 2011 Côte d'Ivoire DHS reports and the 2000, 2006 and 2016 Côte d'Ivoire MICS reports). DHS, Demographic and Health Surveys; INTRO, timely introduction of complementary foods; MAD, minimum acceptable diet; MDD, minimum dietary diversity; MICS, Multi‐Indicator Cluster Survey; MMF, minimum meal frequency.

The proportion of children meeting CF indicators is presented in Figure 1. Overall, the CF practices in Côte d'Ivoire were improved over the past two decades. Pooling data from 1994 to 2016, the average proportion of children aged 6–8 months achieving INTRO was 56.9%. From 2006 to 2016, the proportion of children under 5 years of age meeting INTRO has increased from 39.7% to 65.5%, following a decline from 60.9% to 39.7% in 1994. The proportion of children aged 6–23 months meeting MMF and MDD increased since 2011 when national data became available. From 2011 to 2016, MMF increased from 40.2% to 47.7% and MDD increased from 11.3% to 26.0%. As a composite indicator of MMF and MDD, an increasing trend was observed in the proportion of those meeting MAD; the proportion of children aged 6–23 months meeting MAD increased from 4.6% to 12.5% from 2011 to 2016 (Figure 1).

The independent factors associated with CF indicators among children aged 6–23 months in Côte d'Ivoire are presented in Table 1. At the individual level, child age, maternal educational levels, maternal TV watching and current breastfeeding status were each significantly associated with at least one of four CF indicators. Compared to children aged 6–11 months, children aged 12–17 and 18–23 months had 4.00 (95% confidence interval [CI]: 3.00, 5.33) and 4.41 (95% CI: 3.16, 6.16) times higher odds of meeting MDD, respectively. Compared to children with mothers who received no education, those with mothers who had secondary‐ or higher‐level education were more likely to meet INTRO (2.69 [95% CI: 1.23, 5.91]) and MDD (1.40 [95% CI: 1.00, 1.94]). Maternal TV watching at least once a week compared to no exposure to media was significantly associated with 1.38 (95% CI: 1.06, 1.78) times higher odds of meeting MDD. Compared to children with mothers who were not currently breastfeeding, those with mothers who were currently breastfeeding had 1.48 (95% CI: 1.20, 1.83) times higher odds of meeting MMF, respectively (Table 1). Additionally, older child age and maternal breastfeeding were both significantly predictive of higher odds of meeting MAD. Children aged 12–17 and 18–23 months had 3.41 (95% CI: 2.34, 4.96) and 3.63 (95% CI: 2.35, 5.62) times higher odds of meeting MAD, respectively, compared to children aged younger. Children who were currently breastfed had approximately six times (5.74 [95% CI: 3.49, 9.44]) higher odds of meeting MAD than those who were not receiving breastfeeding. At the household level, compared to children living in urban settings, those from rural households had lower odds of meeting MDD (odds ratio: 0.62, 95% CI: [0.46, 0.85]). In contrast to children from wealthier households, children from poorer households were less likely to meet MMF and MDD; the odds ratios ranged from 0.56 to 0.62. The odds of meeting MAD were significantly predicted by urbanicity and household wealth levels. Children living in rural settings had lower odds (0.65 [0.43, 0.97]) of meeting MAD compared to those residing in urban areas, and children from poorer families had lower odds (ranging from 0.36 to 0.50) of meeting MAD compared to those living in the richest families (Table 1). At the community level, access to health care was significantly associated with meeting MMF. Higher access to health care was adversely associated with meeting MMF (Table 1).

**Table 1:** Multivariate logistic regression results on significant factors (OR and 95% CI) associated with IYCF indicators in children 6‐23 months in Côte d'Ivoire (2016)

		INTRO		MMF		MDD		MAD	
		394		2120		2378		2043	
		OR		OR		OR		OR	
		(95%CI)	*P*‐value	(95%CI)	*P*‐value	(95%CI)	*P*‐value	(95%CI)	*P*‐value
***Child characteristics***									
Age (months)									
	6‐11					1.00		1.00	
	12‐17					**4.00**	**<0.001**	**3.41**	**<0.001**
						**(3.00, 5.33)**		**(2.34, 4.96)**	
	18‐23					**4.41**	**<0.001**	**3.63**	**<0.001**
						**(3.16, 6.16)**		**(2.35, 5.62)**	
***Maternal characteristics***									
Maternal Education									
	No education	1.00				1.00			
	Primary	1.12				1.13			
		(0.68, 1.85)				(0.88, 1.46)			
	Secondary/Higher	**2.69**	**0.01**			**1.40**	**0.05**		
		**(1.23, 5.91)**				**(1.00, 1.94)**			
Breastfeeding practices									
	Currently breastfeeding			**1.48**	**<0.001**			**5.74**	**<0.001**
				**(1.20, 1.83)**				**(3.49, 9.44)**	
Exposure to media at least once a week									
	Watching TV					**1.38**	**0.01**		
						**(1.06, 1.78)**			
***Household characteristics***									
Living in rural areas						**0.62**	**<0.01**	**0.65**	**0.04**
						**(0.46, 0.85)**		**(0.43, 0.97)**	
Household wealth, quintiles									
	1 Richest			1.00		1.00		1.00	
	2			0.88		0.70		**0.45**	**<0.01**
				(0.60, 1.28)		(0.47, 1.04)		**(0.27, 0.74)**	
	3			**0.56**	**<0.01**	**0.62**	**0.03**	**0.36**	**<0.001**
				**(0.38, 0.83)**		**(0.41, 0.95)**		**(0.21, 0.62)**	
	4			0.74		**0.58**	**0.03**	**0.50**	**0.03**
				(0.48, 1.15)		**(0.35, 0.94)**		**(0.27, 0.92)**	
	5 Poorest			**0.56**	**0.01**	0.60		**0.37**	**<0.01**
				**(0.36, 0.89)**		(0.36, 1.01)		**(0.19, 0.72)**	
***Community characteristics***									
	Rank of access to health care, quintiles								
	1 Best access			1.00					
	2			0.98					
				(0.73, 1.31)					
	3			**1.37**	**0.04**				
				**(1.02, 1.85)**					
	4			1.28					
				(0.94, 1.74)					
	5 Worst access			1.07					
				(0.78, 1.47)					

Abbreviations: CI, confidence interval; INTRO, timely introduction of complementary foods; IYCF, Infant and Young Child Feeding; MAD, minimum acceptable diet; MDD, minimum dietary diversity; MMF, minimum meal frequency; OR, odds ratio.

## 4 DISCUSSION

Using nationally representative data in Côte d'Ivoire, we found an improvement in CF practices from 1994 to 2016. Although an overall decreasing pattern was observed in the proportion of infants and young children meeting INTRO before 2006, it has improved from 2006 to 2016. From 2011 to 2016, the proportions of children at 6–23 months of age meeting MMF, MDD and MAD all increased steadily. We also identified influencing factors of meeting at least one of the four indicators, INTRO, MMF, MDD and MAD, including older child age, maternal education levels, maternal TV watching, currently breastfeeding, residing in an urban area, wealthier households and living in communities with better health care access. This study provides a better understanding of CF practices over the past two decades in Côte d'Ivoire and identifies factors that may help determine infants and young children are at risk of experiencing poor CF practices.

Greater investments in multistakeholder, national projects to promote CF practices may have played a role in the upward trends of CF in Côte d'Ivoire between 2011 and 2016. For example, the Ministry of Health (National Nutrition Program and the National Institute of Public Health) developed a national recipe guide based on locally grown and affordable foods to help improve CF in children (Food and Nutrition Technical Assistance [FANTA], 2015). The Global Alliance for Improved Nutrition (GAIN) also implemented a nationwide project in 2009 to introduce nutritious and affordable fortified complementary food and raise awareness of CF practices targeting economically disadvantaged children aged 6–23 months and their caregivers (GAIN, 2014). Despite the investment, the implementation of such programmes, civil wars and continuous conflicts may have mitigated their effectiveness in improving CF practices, threatening child wellbeing and nutrition. Between 2002 and 2008, a period of frequent inner war and conflicts, the poverty rate in Côte d'Ivoire increased by 10 percentage points, from 38.4% to 48.9% (IMF, 2009). This time period coincides with the drop in the prevalence of timely INTRO (from 60.9% to 39.7%), which likely resulted from extreme hardships in food access (Fürst et al., 2010). Although there were steady improvements in the economy after the war, the poverty rate in Côte d'Ivoire remained high (46.3%) in 2015 (The World Bank, 2020a). In poor households, the main barrier to widespread utilization of complementary foods was the cost of the locally produced fortified complementary foods for poor consumers (Leyvraz et al., 2016). Despite the poverty and conflicts caused by the civil war, continuous efforts have been made to improve CF practices by international and national stakeholders representing the United Nations, Government, civil society, and nongovernment organizations. For instance, in 2016, the government allocated a national budget of about 470 million US dollars for a Public Investment Program under the National Multi‐Sectoral Nutrition Plan to improve the nutritional problems within the country (Initiatives, 2020). Although appropriate CF practices remain low in Côte d'Ivoire, the net positive CF trends and continued investments in nutrition programming are encouraging.

In previous studies in other low‐income countries where food access is not guaranteed (Mitchodigni et al., 2017; Taha et al., 2020), older children within the 6–23 age range were more likely to be meeting MDD and MAD. A common feeding pattern has been observed in Sub‐Saharan African countries, where young children's diets are mostly comprised of grains, roots, and tubers with little variety (Faber, 2005; Macharia‐Mutie et al., 2010; Maseta et al., 2008; Sika‐Bright, 2010), and to a lesser extent, fruits, vegetables and animal‐sources foods, which tend to be consumed as children grow older (Faber, 2005). Another possible explanation proposed by Ali et al. is that limited maternal literacy or awareness levels of feeding practices may also result in poor dietary diversity in older children (Ali et al., 2021). Our study findings on higher maternal education levels associated with higher odds of meeting MDD might support Ali's explanation. As children grew older, rural mothers with lower literacy and awareness levels were more likely to feed their children based on children's demands on certain types of food rather than feeding diverse food types (Ali et al., 2021). However, the MICS 2016 survey still lacked data on maternal awareness levels on child feeding; therefore, future studies are needed to further investigate Ali et al.'s explanation using national‐level data in Côte d'Ivoire.

In addition to the child's age and maternal education levels, maternal TV watching, and whether mothers were currently breastfeeding their children were significantly associated with CF practices in Côte d'Ivoire. Maternal media exposure has been identified as a key modifiable risk factor associated with improved CF practices in multiple countries (Beyene et al., 2015; Joshi et al., 2012; Malhotra, 2013). Mass media in Côte d'Ivoire has become an important platform to deliver appropriate feeding information. For example, national TV programmes have broadcast innovative media campaigns to promote the feeding of complementary foods (Hystra, 2014). Caregivers may improve their awareness of child feeding by receiving educational health messages channelled through media (Patel et al., 2012), and educating caregivers on child feeding through mass media is considered acceptable and feasible (Rahman et al., 2012). Besides, owning a TV in the household may be a proxy indicator of household wealth levels, and our findings showed that children from wealthier households had higher odds of meeting all four CF indicators compared to those from poorer households.

Continued breastfeeding was another significant modifiable risk factor influencing multiple CF indicators, including MMF and MAD. Our finding is consistent with previous evidence from 367 children aged 6–23 months in Ethiopia, where they found that currently breastfed children were 7.5 times more likely to achieve MMF compared to nonbreastfed children (Wagris et al., 2019). Results from a cross‐sectional study in the United Arab Emirates also showed that 47.3% of breastfed versus 21.9% of nonbreastfed children met MMF among 931 children aged 6–23 months (Taha et al., 2020). Breastfed children compared to nonbreastfed children are more likely to meet the criterion for MMF as their requirements for minimum meal frequency are lower (WHO, 2010).

Several modifiable household and community characteristics were identified as additional barriers to optimal CF practices, especially MDD. At the household level, not living in an urban area was associated with lower odds of meeting MDD and MAD. In Côte d'Ivoire, approximately equal proportions of the population live in rural and urban areas (The World Bank, 2020b) but the poverty rate in the rural areas is much higher (57% vs. 36%) (The World Bank, 2018). Previous formative research also identified limited caregiver knowledge and restricted household access to nutritious and affordable complementary food options as barriers to optimal CF in rural areas (Recherche formative sur les pratiques d'alimentation du nourrisson et du jeune enfant [REFACE] PACE/IYCN, Rapport final., 2010). These findings may further explain the lower odds of meeting MDD and MAD among children in rural areas. We observed lower odds of meeting MMF, MDD and MAD in poorer households compared to wealthier households. In all countries, especially those in postconflict countries such as Côte d'Ivoire, poverty has been identified as a major barrier to appropriate CF practices (Nassanga et al., 2018). In 2015, approximately half of all households were under the national poverty line in Côte d'Ivoire (The World Bank, 2020a). Echoing prior findings, compared to children from nonpoor households (multidimensional poverty index ≥0.33), a lower proportion of children from poor households met age‐appropriate dietary diversity (65.9% and 51.9%, respectively) in Côte d'Ivoire (Leyvraz et al., 2016). Taken together, infants and young children from poor and rural households appear to be particularly vulnerable to risks associated with not meeting CF and interventions that provide food and/or financial resources may be helpful. Expanding nutrition programme coverage through enhanced political will and sustained investments to scale up programming is critical for ensuring that the most vulnerable infants and young children have their health and nutrition needs met in Côte d'Ivoire.

Our study had several important strengths, including the use of nationally representative survey data, standardized measurement instruments and procedures from DHS and MICS surveys, and well‐designed analytical methods based on a conceptual framework and previously published work. However, our study also has some limitations. The nature of cross‐sectional surveys prevents us from determining causality. The use of self‐reported data may raise the possibility of recall bias, as well as respondent bias if mothers respond to survey questions based on perceived interviewers' desirability. Another limitation is the lack of consideration for other possible predictors that might affect the CF practices, such as food availability and allocation of food and financial resources within each household (United Nations Children's Fund, 2020a).

## 5 CONCLUSION

CF practices among children aged 6–23 months remain poor in Côte d'Ivoire. Although an improvement in CF practices has been observed among infants and young children since 2006, only about 13% of children met the MAD criterion. Among all CF feeding indicators, dietary diversity remains a major challenge among infants and young children in Côte d'Ivoire. Improvement in identified modifiable influencing factors at the individual, household and community levels. Understanding the crucial roles of modifiable risk factors at the individual, household and community levels may greatly influence the CF practices in young children. Evidence‐based, multi sectorial strategies aiming at improving CF practices should focus on the most vulnerable infants and young children in rural and poor households where health access may be limited. As nations such as a Côte d'Ivoire work in multi‐sectoral partnerships to reach Sustainable Development Goal #2: *Zero Hunger*, increased political will, enhanced investments, and greater collaborations are needed to help fill important nutrient gaps of infants and young children through the *First 1,000 Days* of life.

## AUTHOR CONTRIBUTIONS

Muzi Na, Stephen R. Kodish, Laura E. Caulfield and Laura E. Murray‐Kolb designed the research study. Muzi Na and Nan Dou analyzed the data. Nan Dou wrote the manuscript. Muzi Na, Stephen R. Kodish, Laura E. Caulfield, Laura E. Murray‐Kolb, Nan Dou, Evaniya Shakya, Raphia M. Ngoutane, Denis Garnier, Oka R. Kouame, Anne‐Sophie L. Dain and Aashima Garg reviewed and edited the manuscript. All authors contributed to the article and approved the submitted version.

## CONFLICT OF INTEREST

The authors declare no conflict of interest.

## Data Availability

The data that support the findings of this study are openly available from the DHS programme website at https://dhsprogram.com/data/available-datasets.cfm, and the MICS programme website at https://mics.unicef.org/surveys.
